# CCL22-producing macrophages are associated with Th1-related sweat duct inflammation in acquired idiopathic generalized anhidrosis

**DOI:** 10.3389/fimmu.2026.1831853

**Published:** 2026-05-08

**Authors:** Shingo Takei, Ryota Hayashi, Manon Okamura, Tatsuya Katsumi, Toru Kawai, Riichiro Abe

**Affiliations:** Division of Dermatology, Niigata University Graduate School of Medical and Dental Sciences, Niigata, Japan

**Keywords:** acquired idiopathic generalized anhidrosis, alopecia areata, anhidrosis, CCL22, IFN-γ

## Abstract

**Background:**

Acquired idiopathic generalized anhidrosis (AIGA) is a rare disorder characterized by generalized loss of sweating without identifiable causes. Because few biomarkers reflect its underlying mechanisms, diagnosis at the initial visit is often difficult. Although steroid pulse therapy is widely used, approximately half of patients respond insufficiently. We therefore aimed to elucidate the immune mechanisms underlying AIGA and identify potential biomarkers for diagnosis and treatment response.

**Methods:**

Fourteen patients with AIGA affecting more than 25% of body surface area were enrolled after exclusion of secondary causes of anhidrosis. Serum levels of 40 cytokines and chemokines were quantified using a multiplex assay and correlated with clinical parameters. Skin biopsy specimens were analyzed by histology and immunohistochemistry to characterize inflammatory cell infiltration and identify cellular sources of selected mediators.

**Results:**

Inflammatory cell infiltration was consistently observed around sweat ducts, predominantly composed of CD4^+^ T cells. Serum profiling revealed significant elevations of CCL22 and IFN-γ in AIGA compared with healthy controls, with a strong positive correlation between them. Consistently, the downstream chemokine CXCL10 was also increased. Double immunostaining identified CD68^+^ macrophages as the main source of CCL22 in periductal regions. Serum Macrophage migration inhibitory factor (MIF) levels were significantly higher in steroid-resistant cases, whereas MIF expression within sweat ducts was markedly reduced, suggesting disruption of local immune privilege.

**Conclusions:**

These findings suggest that AIGA involves a macrophage–CCL22–Th1–IFN-γ inflammatory axis associated with collapse of sweat duct immune privilege. Serum MIF may serve as a potential biomarker for predicting steroid responsiveness.

## Introduction

Acquired idiopathic generalized anhidrosis (AIGA) is a sweating abnormality resulting in generalized anhidrosis or hypo-sweating with no apparent cause identified (1). Patients with AIGA frequently suffer from heat intolerance due to impaired thermoregulation, significantly declining their quality of life. AIGA is diagnosed by excluding recognized causes of anhidrosis, such as neurological, endocrine, or metabolic disorders including Fabry’s disease, and by documenting decreased sweating over more than 25% of body surface area using assessing tools such as the Minor’s starch-iodine test (1). However, even if decreased sweating is confirmed by Minor’s test, it is challenging to fully exclude underlying diseases at the initial presentation based on routine assessments. Currently, while increases in ductal markers such as serum CEA have been described (2), disease-specific biomarkers that could aid in diagnosis remain largely unestablished, highlighting the necessity for their development. In patients with AIGA, steroid pulse therapy is often administrated based on the hypothesis that autoimmunity against sweat ducts/glands plays a role in the pathogenesis (3). However, this treatment approach remains empiric, and 43% of patients has been reported to fail to respond (4). The lack of reliable predictors for treatment responsiveness and the unclear immunopathological mechanisms of the disease have hampered the development of targeted therapies. Although the precise pathomechanisms underlying AIGA remain largely unknown, previous reports have described periductal infiltration of CD3^+^ cytotoxic T cells in affected skin, suggesting a T cell–mediated autoimmune response directed at the eccrine sweat ducts (5). Interestingly, this pathological feature shares similarities with autoimmune disorders such as alopecia areata (AA), in which collapse of immune privilege and T cell–mediated attack against hair follicles are centers of the pathogenesis (6). These parallels raise the possibility that AIGA may also involve localized immune dysregulation targeting ductal epithelia. Given these observations, our study focused on chemokines as potential contributors to the immune-mediated pathogenesis of AIGA, since previous reports have described inflammatory cell infiltration around the sweat ducts and chemokines are key mediators responsible for directing the migration of such immune cells. Therefore, we conducted a comprehensive analysis of serum chemokines in patients with AIGA to gain insight into disease mechanisms and to identify potential diagnostic or therapeutic biomarkers.

## Methods

### Patients

Serum samples and skin biopsy specimens from anhidrotic areas of patients with AIGA (n=14) at our institution were included. The diagnosis of AIGA was made based on the following criteria in accordance with Japanese guideline (1): (1) absence of known causes of anhidrosis, including endocrine disorders, neurological diseases based on physical examination and routine blood tests; and (2) presence of generalized anhidrosis affecting more than 25% of the body surface area (BSA), as evaluated using the Minor’s starch-iodine test. The severity of AIGA was classified according to the Japanese severity grading system; mild: <50% of BSA affected; moderate: ≥50% to <75%; severe: ≥75%. Serum samples from healthy volunteers without any dermatological disorders and from patients with AA (n=16) were also collected as healthy controls (HCs) (n=8) and disease comparator groups, respectively. None of the patients with AIGA included in this study had concomitant AA or clinically evident hair loss. The alopecia areata (AA) cohort included patients with a range of clinical subtypes (multiplex, totalis, and universalis) and disease stages (progressive or stable). This approach was intended to reflect the heterogeneity of the AIGA cohort, which also included patients with variable disease duration, extent of anhidrotic body surface area, and treatment status. Correlation analyses were performed between selected chemokines and clinical parameters, including disease duration (period from onset to treatment), severity (based on BSA), and treatment responsiveness.

### Treatment and evaluation of steroid responsiveness

In patients with AIGA treated with steroid pulse therapy, intravenous methylprednisolone at 1,000 mg/day for 3 consecutive days was administered. Responsiveness to therapy was defined based on the number of courses required to achieve improvement in sweating, which was determined when patients themselves clearly recognized a recovery of generalized sweating. Patients requiring three or more courses were classified as treatment-resistant. This definition is supported by a previous report showing that 73% (n=59/80) of patients with AIGA who responded to steroid pulse therapy demonstrated clinical improvement within two courses of treatment (4).

### Comprehensive Serum Chemokine Analysis and ELISA Validation

The serum levels of 40 chemokines and chemotaxis-related cytokines were measured using the Human Chemokine 40-plex Panel (Bio-Rad Laboratories, Hercules, CA, USA) according to the manufacturer’s instructions. Peripheral blood samples were collected and processed on the same day to minimize pre-analytical variability. After collection, samples were centrifuged at 3,000 rpm for 10 minutes, and the resulting serum was carefully collected. The serum was aliquoted into 100 μL portions to avoid repeated freeze–thaw cycles and stored at −80 °C until analysis. Immediately prior to multiplex analysis, serum samples were thawed on ice and centrifuged at 10,000 rpm for 10 minutes at 4 °C to remove particulate material. The clarified supernatant was used for all measurements. Data were analyzed by measuring fluorescent beads using a Bio-Plex 200 system (Bio-Rad Laboratories). Standards and serum samples were measured in duplicate. Values below the detection limit were rated as zero and those above were rated as the value of the detection limit. Data analysis was performed using Bio-Plex Manager Software (V6.1; Bio-Rad Laboratories) to calculate concentrations in pg mL−1.

Serum CCL22 levels were quantified using a Human CCL22/MDC Quantikine ELISA Kit (DMD00, R&D Systems, Minneapolis, MN, USA) according to the manufacturer’s instructions. All samples were measured in duplicate, and the mean values were used for analysis. Concentrations were calculated using standard curves generated for each assay.

### Histopathological evaluation with immunohistochemistry

Formalin-fixed, paraffin-embedded (FFPE) tissue sections were stained with hematoxylin and eosin (H&E) to evaluate the distribution and extent of inflammatory cell infiltration around sweat ducts and sweat glands. Immunohistochemical staining of FFPE sections was performed according to standard procedures. Sections were incubated overnight at 4 °C with primary antibodies against CD3 (polyclonal, Aglient Technologies, Santa Clara, CA, USA), CD4 (clone 1F6, Nichireibiosciences, Tokyo, Japan), CD8 (clone C8/144B, Nichireibiosciences), CD20 (clone L26, Aglient Technologies), CD68 (clone KP1, Aglient Technologies), CCL22 (clone EPR1362, Abcam, Cambridge, UK), CCR4 (clone F9A9K, Cell signaling Technology, USA) and MIF (polyclonal, Atlas Antibodies, Bromma, Sweden), followed by incubation with HRP-conjugated secondary antibodies (Envision+System-HRP-labelled polymer antirabbit or mouse);(Aglient Technologies). Signals were visualized using DAB substrate, and hematoxylin was used for nuclear counterstaining. For immunofluorescence staining, FFPE sections were deparaffinized, and primary antibodies against CCL22 (clone EPR1362, Abcam, Cambridge, UK) and CD68 (clone KP1, Aglient Technologies) were applied overnight at 4 °C, followed by Alexa Fluor–conjugated secondary antibodies. Nuclei were counterstained with DAPI.

### Flow cytometric analysis

PBMCs were isolated from whole blood by density gradient centrifugation using Ficoll-Paque (GE Healthcare, Chicago, IL, USA) according to the manufacturer’s instructions. CD4^+^ T cells were then isolated from PBMC of patients with AIGA and HCs using CD4^+^ T cell Isolation Kit (Miltenyi Biotec, Bergisch Gladbach, Germany) and were cultured with phorbol myristate acetate (PMA; 50ng/mL, Sigma-Aldrich, St. Louis, MO, USA) and ionomycin (IM; 1μg/mL, Sigma-Aldrich) in the presence of Brefeldin A (eBioscience, San Diego, CA, USA) for the last 6 hours. After incubating, cells were stained with an PE-Cy7-conjugated anti-CD3 and FITC-conjugated anti-CD4 mAbs, fixed in fixation/permeabilization solution (eBioscience); permeabilized in permeabilization buffer (eBioscience); and stained with PE-anti-IFN-γ mAb. BD FACSLyric flow cytometer (BD Bioscience, San Jose, CA, USA) was used to identify the cell population and intracellular cytokine expression.

### Statistical analysis

Statistical analysis was performed using Prism software. Statistical comparisons between two groups (AIGA vs HCs) and among three groups (AIGA vs AA vs HCs) were performed using the Mann–Whitney U test and the Kruskal–Wallis one-way analysis of variance, respectively. To account for multiple testing, p values were adjusted using the Benjamini–Hochberg false discovery rate method. Correlations between chemokine levels were evaluated using Spearman’s rank correlation coefficient. Differences were considered statistically significant at *P* < 0.05.

### Ethics statement

This study was approved by the Human Research Ethics Committees of Niigata University Medical and Dental Hospital (approval number:2023-0227, 2023-0282, 2024-0274).

## Results

### The clinical characteristics of patients with AIGA

Fourteen patients with AIGA were included to this analysis. All 14 patients were male, with a mean age of 31 years (standard deviation, 14.8 years; age range, 14–59 years) (**Table 1**), which is consistent with previous reports describing a predilection for younger male patients (1). The prevalence of cholinergic urticaria among these patients was notably high at 79% (11 out of 14 cases) (**Table 1**), as previously documented (7). The severity of AIGA in each patient was classified based on affected BSA (%) according to the Japanese severity grading system. As a result, 2 patients were classified as having mild disease, 3 as moderate, and 9 as severe (**Table 1**). Steroid pulse therapy was predominantly employed for treating these AIGA patients (13 out of 14 cases, 93%). The median duration from disease onset to the initiation of steroid pulse therapy was 5.5 months (range, 1–84 months). (**Table 1**). Among the patients who received steroid pulse therapy (n=13), 61% (n=8) responded positively, 31% (n=4) were resistant who were required more than 3 treatment courses, and treatment efficacy was unknown in 8% (n=1) (**Table 1**).

**Table 1:** Clinical characteristics of patients with AIGA.

Patient	Age	Sex	BSA (%) of anhidrotic area	Severity	Period to treatment (month)	Resistance to treatment	Cholinergic urticaria	Inflammatory cell infiltration of sweat glands	
								Periductal area	perisecretory area
1	29	M	40	mild	84	+	+	+	–
2	50	M	40	mild	N/D	N/D	+	N/A	N/A
3	14	M	70	moderate	8	–	–	+	–
4	30	M	70	moderate	5	+	+	+	–
5	44	M	70	moderate	8	unknown	+	+	–
6	52	M	75	severe	1	–	+	N/A	N/A
7	14	M	90	severe	7	–	+	+	–
8	16	M	90	severe	7	+	+	+	+
9	30	M	90	severe	3	–	–	+	–
10	32	M	90	severe	2	–	+	+	+
11	19	M	≧95	severe	6	–	+	+	–
12	22	M	≧95	severe	4	–	+	+	–
13	23	M	≧95	severe	1	+	+	+	–
14	59	M	≧95	severe	12	–	–	+	–

### Preferential T-cell infiltration around sweat ducts in AIGA

To evaluate inflammatory cell infiltration around sweat ducts and sweat glands, H&E staining was performed on skin biopsy specimens obtained from the anhidrotic areas of patients with AIGA (n = 12). In all cases, inflammatory cell infiltration was observed around the sweat ducts, whereas infiltration around the secretory portions of the sweat glands was detected in only two cases (**Table 1A**), indicating that inflammation preferentially occurs around the sweat ducts rather than the secretory portions of the sweat glands, which is consistent with previous reports (5).

**Figure 1:**
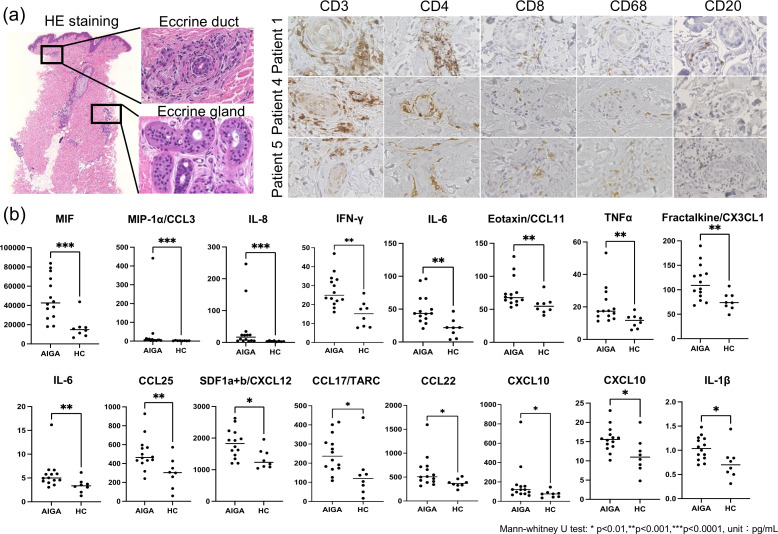
**(A)** Representative histopathological and immunohistochemical findings of skin lesions in patients with AIGA. Hematoxylin–eosin staining shows dense inflammatory cell infiltration predominantly around the eccrine ducts but not the secretory coils. Immunohistochemistry demonstrates that infiltrating cells are mainly CD3^+^ T cells, particularly CD4^+^ cells, with fewer CD8^+^ T cells and CD68^+^ macrophages. CD20^+^ B cells are rarely observed. **(B)** Comprehensive serum chemokine profiling in patients with AIGA (n=14) and healthy controls (HCs)(n=8). Among chemokines with values within the measurable range, 16 chemokines/cytokines were significantly elevated in AIGA compared with HCs. Data are presented as median with interquartile range. Mann–Whitney U test: *p<0.05, **p<0.01, ***p<0.001. Schematic illustration was created with BioRender.com.

Subsequently, to characterize the types of inflammatory cells infiltrating the periductal region, immunohistochemical staining for CD3, CD4, CD8, CD20, and CD68 was performed. As a result, the inflammatory cells infiltrating around the sweat ducts were predominantly CD3-positive T cells, with a minor population of CD68-positive histiocytes, while CD20-positive B cells were rarely observed (**Figure 1A**). Among the T cells, CD4+, CD68- T helper (Th) cells were more abundant than CD8-positive cells (**Figure 1A**).

### Comprehensive chemokine profiling in the serum of patients with AIGA

We next investigated chemokines that may contribute to the recruitment of inflammatory cells around sweat ducts. We instead measured serum levels of 40 chemokines and chemotaxis-related factors using the Human Chemokine Panel 40-plexⓇ (Bio-Rad) in patients with AIGA (n = 14) and HCs (n = 8). As a result, among chemokines with values within the measurable range, 16 chemokines/cytokines were found to be significantly elevated in the serum of patients with AIGA compared to healthy controls. These chemokines and cytokines included MIF, CCL3, CCL22, CCL25 and IL-1β as macrophage-derived factors; IFN-γ and TNF-α, IL-6 as proinflammatory cytokines; IL-8 as a neutrophil-associated chemokine; and IL-16, Eotaxin/CCL11, and TARC/CCL17 as mediators associated with Th2-type inflammation; and IL-10 as anti-inflammatory cytokine; and CXCL3CL1, CXCL12, and CXCL10 as other chemotactic factors (**Figure 1B**; **Supplementary Table S1**; **Supplementary Figure 1**). After correction for multiple comparisons, above chemokines remained significantly elevated in AIGA (**Supplementary Figure 1**).

### Elevated serum MIF levels and decreased local expression in sweat ducts

**Figure 2:**
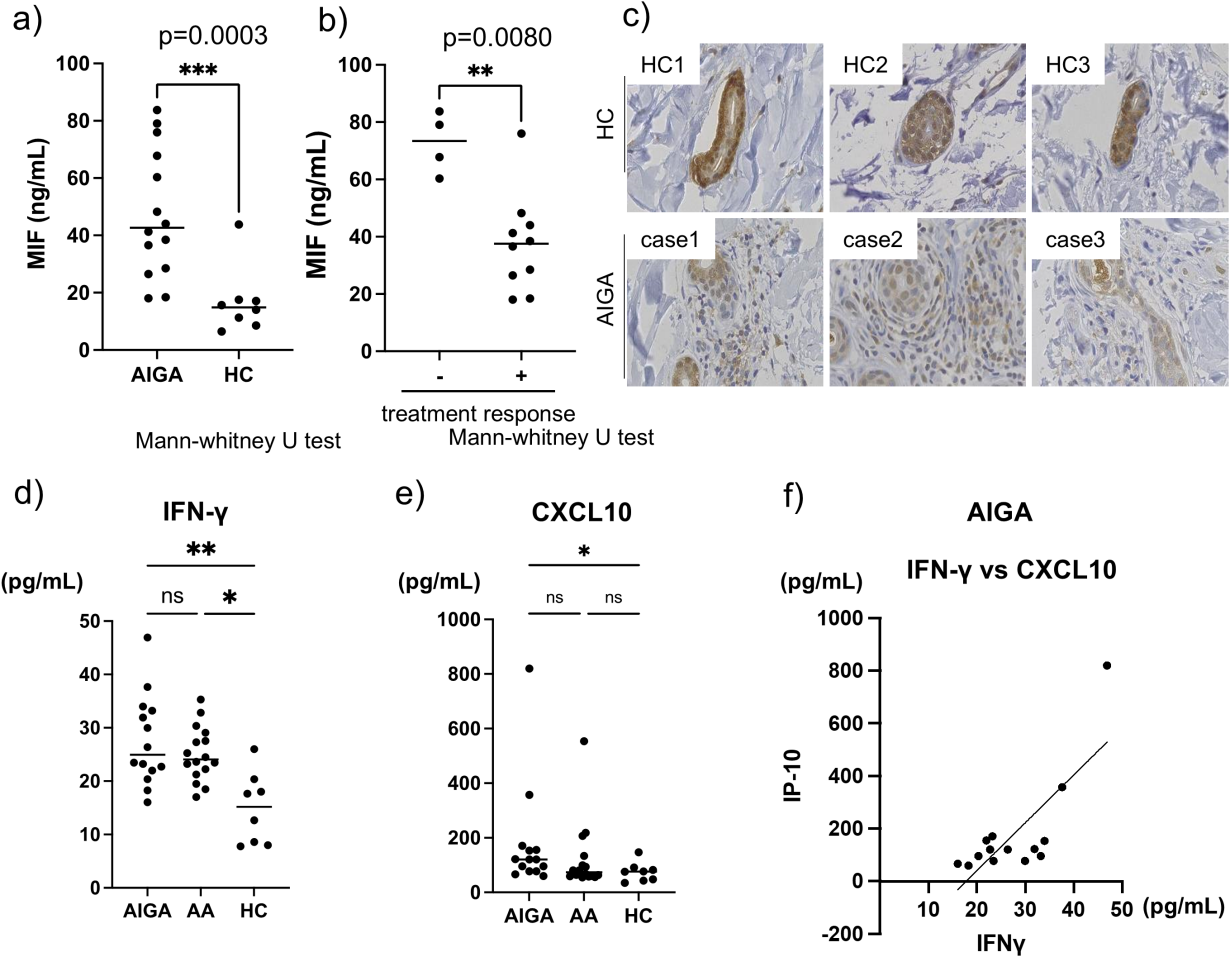
**(A–C)** Serum MIF levels and local expression in sweat ducts. **(A)** Serum MIF levels were significantly elevated in AIGA (n=14) compared with HCs (n=8). Mann–Whitney U test: ***p<0.001. **(B)** Serum MIF levels were significantly higher in treatment-resistant patients (n=4) compared with treatment-responsive patients (n=10). Mann–Whitney U test: **p<0.01. **(C)** Immunohistochemistry reveals decreased MIF expression in eccrine ducts from AIGA skin compared with HCs. **(D–F)** Serum IFN-γ and CXCL10 levels in AIGA (n=14), alopecia areata (AA) (n=16), and HCs (n=8). **(D, E)** Serum IFN-γ and CXCL10 levels are significantly higher in AIGA than in HCs. Mann–Whitney U test: *p<0.05, **p<0.01. **(F)** A significant positive correlation between IFN-γ and CXCL10 levels is observed in AIGA. Spearman’s test: p<0.05.

Among the chemokines elevated in AIGA, we focused on macrophage migration inhibitory factor (MIF), which showed the most significant difference between AIGA and HCs (AIGA 42.7 vs 14.9 ng/mL, p=0.0003) (**Figure 2A**). MIF is involved in various function including leukocyte recruitment, or inflammation and its serum levels are elevated in sepsis and autoimmune diseases such as systemic lupus erythematosus (SLE) (8). We first investigated whether serum MIF levels correlated with clinical features of AIGA. Although no correlation was observed between serum MIF levels and disease severity or duration, patients with poor responsiveness to steroid pulse therapy exhibited significantly higher serum MIF levels compared to those with good responsiveness (73.4 vs 37.5ng/mL, p=0.008) (**Figure 2B**; **Supplementary Figure 2**). It has also been reported that MIF may play a role in the formation of steroid resistance in systemic lupus erythematosus by affecting the NF-κB/IκB signaling cascade and is considered to be a potential target for steroid sparing (9). These findings suggest that MIF may also serve as a potential indicator of steroid resistance in AIGA. We next hypothesized that high serum MIF levels may reflect increased local expression of MIF in sweat ducts, which in turn may contribute to the recruitment of inflammatory cells around the eccrine ducts. To test this hypothesis, we performed immunohistochemical staining for MIF in skin biopsy specimens from patients with AIGA. As a result, contrary to our expectations, MIF expression in the sweat ducts of patients with AIGA was decreased compared to that in HCs (**Figure 2C**). Reduced MIF expression has been reported in hair follicles in AA and in sweat glands in Sjögren’s syndrome (6, 10). *In vitro* studies have shown that MIF can suppress NK cell-mediated cytotoxicity in AA, and in Sjögren’s syndrome, an inverse correlation has been observed between MIF expression in sweat ducts and the extent of surrounding inflammatory cell infiltration (6, 10). There have been reports that MHC class I expression, which is normally downregulated to help evade immune responses, is upregulated in the sweat ducts of AIGA (11). Taken together with the observed reduction of MIF expression in the ducts, these findings strongly suggest that a breakdown of immune privilege is likely involved in the pathogenesis of AIGA.

### IFN-γ-associated immune responses and CCL22^+^ macrophage in AIGA

**Figure 3:**
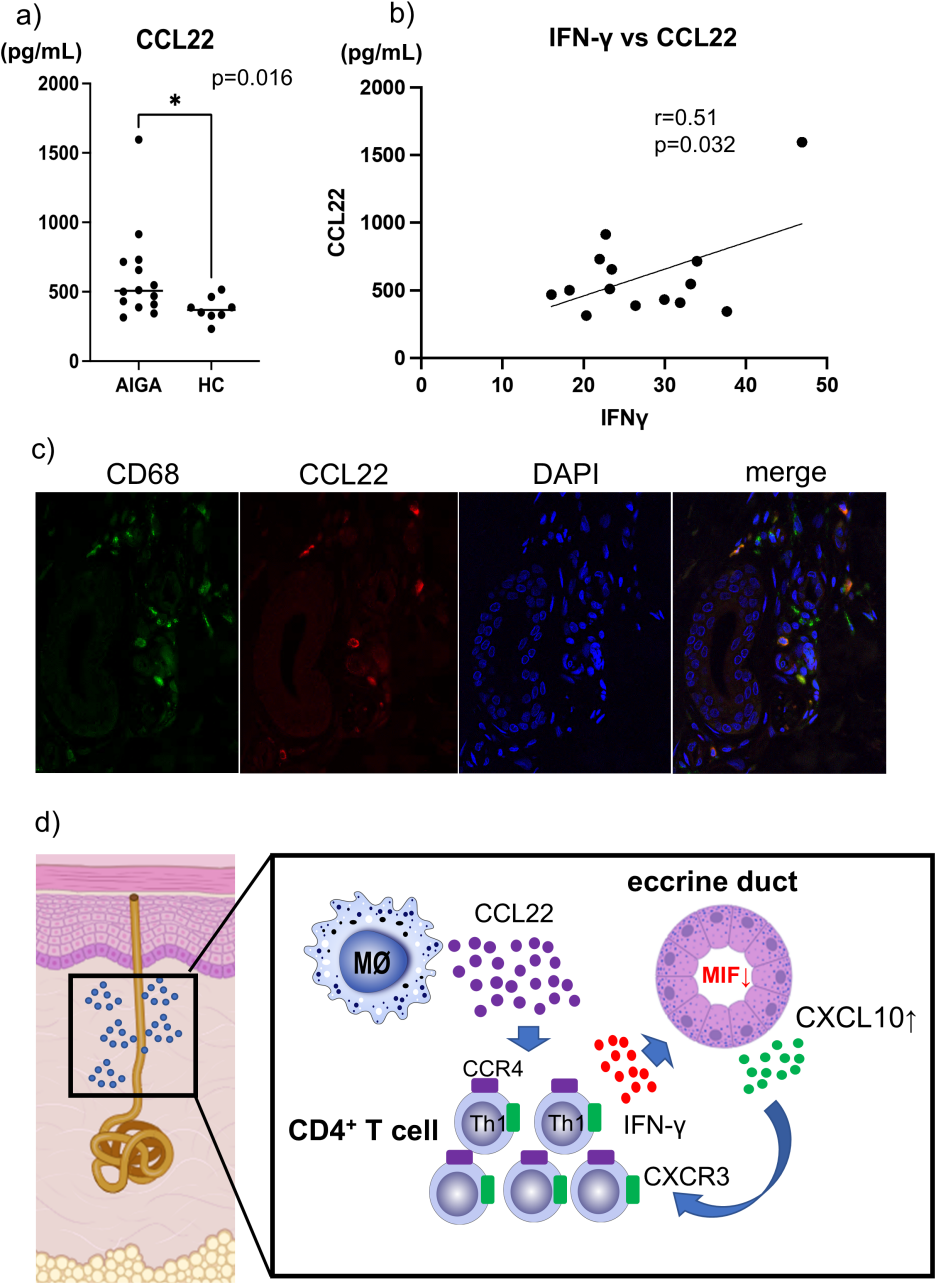
Serum CCL22 levels, correlation with IFN-γ, and identification of CCL22-expressing cells. **(A)** Serum CCL22 levels are significantly higher in AIGA (n=14) than in HCs (n=8). Whitney U test: *p<0.05. **(B)** Serum CCL22 levels are positively correlated with IFN-γ levels in AIGA. Spearman’s test: p<0.05. **(C)** Double immunofluorescence shows CCL22 expression (green) in CD68^+^ macrophages (red) infiltrating around eccrine ducts. Nuclei are counterstained with DAPI (blue).

Next, we hypothesized that a breakdown of immune privilege in sweat ducts, similar to that in AA may lead to autoimmune-mediated eccrine duct damage by self-reactive T cells in AIGA. Previous studies have reported periductal infiltration of cytotoxic T cells expressing TIA-1 and CXCR3, along with high expression of myxovirus resistance protein A (MxA), an interferon-inducible protein that serve as a marker of type I interferon activity, in sweat ducts. These findings suggests that type I interferon signatures, such as IFN-α and IFN-β, may be involved in the pathogenesis (5). Based on our chemokine/cytokine analysis, we noted elevated levels of IFN-γ and its downstream effector CXCL10, an IFN-γ-inducible chemokine that promotes T-cell raising the possibility that IFN-γ, a type II interferon, may also contribute to sweat duct injury in AIGA (**Figure 1B**). We compared the levels of these chemokines between patients with AIGA and those with AA focusing on the shared feature of immune privilege collapse. Serum chemokine levels of 16 patients with AA in both the acute and chronic phases of disease were measured using same methodology as applied to patients with AIGA (**Supplementary Table 2**). As a result, both IFN-γ and CXCL10 were significantly elevated in patients with AIGA, to a degree comparable to that observed in AA, when compared to HCs (IFN-γ: AIGA 23.5 vs HCs 7.81 pg/mL, p=0.0042, CXCL10: AIGA 25.1 vs HCs 13.2 pg/mL, p=0.049) (**Figures 2D, E**). Moreover in AIGA, a significant positive correlation was observed between serum IFN-γ levels and CXCL10 levels (r=0.77, p=0.0006) (**Figure 2F**). Next, we considered CD4^+^ Th1 cells as the main source of IFN-γ production in AIGA. This hypothesis was based on the prominent infiltration of CD4^+^ T-cells around the sweat ducts in AIGA, as well as the fact that Th1 cells are a major cellular source of IFN-γ (12). Indeed, we found that the proportion of CD4^+^ T cells producing IFN-γ was increased in a patient with AIGA, compared to HC, as assessed by intracellular cytokine staining using flow cytometry. Specifically, 5.54% of CD4^+^ T cells from a AIGA patient expressed IFN-γ, whereas only 3.58% were positive in HC (**Supplementary Figure 3**). We next hypothesized that macrophage may contribute to Th1 recruitment, as we noted that CD68^+^ macrophages were present in preductal regions as levels comparable to CD8^+^ T cells, which have already been implicated in the pathogenesis of AIGA. Therefore, we focused on CCL22, a chemokine known to recruit Th1 cells via the CCR4 receptor and to be produced mainly by macrophages. The serum CCL22 levels were significantly elevated in AIGA compared to HCs (506.3 vs 368.9 pg/mL, p=0.016) (**Figure 3A**). To validate the multiplex findings, serum CCL22 levels were additionally measured by ELISA, which confirmed significant elevation in AIGA compared with healthy controls (**Supplementary Figure 4**). Furthermore, significant positive correlations were observed between CCL22 and IFN-γ, as well as between IFN-γ and CXCL10 (r=0.51, p=0.032) (**Figure 3B**). By performing double immunostaining, we identified that the CCL22-expressing cells in AIGA were all CD68^+^ macrophages infiltrating around the sweat ducts and nearly half of macrophage express CCL22 (**Figure 3C**). CCL22-positive macrophages tended to be more abundant in anhidrotic skin, particularly around inflamed eccrine ducts, compared with sweating skin. In addition, CCR4, the receptor for CCL22, was expressed in infiltrating inflammatory cells within these anhidrotic regions, suggesting a potential interaction between CCL22 and CCR4 (**Supplementary Figure 5**). To further evaluate the clinical relevance of the identified chemokines, we performed correlation analyses between selected chemokines (CCL22, IFN-γ, and CXCL10) and clinical parameters. However, no significant correlations were observed between these chemokines and the examined clinical variables (**Supplementary Table S3**). These findings suggest that, although these chemokines are involved in the immunopathological mechanisms of AIGA, their serum levels may not directly reflect disease severity or duration.

## Discussion

Acquired idiopathic generalized anhidrosis (AIGA) is a rare and poorly understood condition characterized by generalized loss of sweating without an identifiable cause. Although the pathogenesis remains unclear, increasing evidence suggests that an immune-mediated mechanism targeting the sweat ducts plays a central role. However, no previous studies have performed comprehensive chemokine or cytokine assays in AIGA, and the underlying immune profile remains unknown. In this study, we investigated serum chemokine profiles and cellular mechanisms underlying sweat duct inflammation, identifying a potential macrophage–Th1 axis mediated by CCL22 and IFN-γ as a key driver of disease pathology (**Figure 3D**).

In our study, a comprehensive serum chemokine profiling revealed significantly elevated levels of IFN-γ and its downstream effector chemokine, CXCL10, in patients with AIGA. Moreover, we observed a strong positive correlation between IFN-γ and CXCL10 concentrations, highlighting the potential contribution of type II IFN (IFN-γ)–mediated immune responses in this disease. This expands the current understanding of interferon signatures in AIGA, suggesting that not only type I IFN previously reported, but also IFN-γ–dependent pathways may be critically involved in sweat duct inflammation and injury. Immunohistochemical analysis of affected skin further demonstrated that CD4^+^ T cells constitute the predominant lymphocytic infiltrate surrounding the sweat ducts. Given that Th1 cells are the principal source of IFN-γ, these findings collectively support a model in which periductal Th1 cell–dominated inflammation drives a IFN-γ–CXCL10 axis.

A novel and important observation in our study was the significant elevation of serum CCL22 and its correlation with IFN-γ levels. CCL22 is classically associated with Th2 cell recruitment via CCR4, but recent evidence also implicates its involvement in promoting Th1 responses under certain inflammatory conditions (13). Notably, a study using a murine model of Sjögren’s syndrome demonstrated that tissue-resident CD11b^high^ macrophages in the salivary glands robustly expressed CCL22, which not only enhanced the migration of CCR4-expressing CD4^+^ T cells but also promoted their IFN-γ production (14). Furthermore, anti-CCL22 antibody administration in that model suppressed local T cell infiltration and ameliorated autoimmune lesions, suggesting that CCL22-producing macrophages contribute to a breakdown of immune tolerance in target tissues (14).

In line with these findings, our double immunofluorescence analysis revealed that CCL22-producing cells in the periductal area of AIGA skin were CD68^+^ macrophages, indicating a similar pathogenic mechanism. These findings suggest that CCL22-producing macrophages may contribute to the recruitment of T cells and the amplification of IFN-γ–associated inflammation.

Interestingly, beyond the CCL22–Th1 axis, our study also implicates the involvement of macrophage migration inhibitory factor (MIF) in the disruption of local immune homeostasis in AIGA. Although serum MIF levels were elevated in a subset of patients with poor steroid responsiveness, we observed a paradoxical reduction in MIF expression within the sweat ducts of affected skin. This finding mirrors prior observations in Sjögren’s syndrome and AA, in which reduced MIF expression in target epithelial structures was associated with increased immune cell infiltration and local tissue destruction (6, 10). Given that MIF can suppress natural killer cell–mediated cytotoxicity and maintain immune privilege in peripheral tissues, its downregulation may facilitate T cell–driven autoimmune injury in sweat ducts, analogous to the collapse of immune privilege described in AA (6).

Consistent with this hypothesis, our comprehensive chemokine profiling revealed that both IFN-γ and CXCL10 were significantly elevated in AIGA patients at levels comparable to those observed in AA. These findings reinforce the concept that AIGA and AA share immunopathological features, including type I and II interferon signatures, impaired epithelial immune privilege, and T cell–mediated epithelial destruction. On the other hand, to our knowledge, there have been no reports of their concomitant occurrence, which may reflect differences in target antigens or immune specificity between the two conditions. Taken together, our data suggest that AIGA may represent a cutaneous autoimmune disorder characterized by localized immune privilege collapse.

This study has several limitations. First, due to the nature of disease, the sample size was relatively small and the study was conducted at a single center, making it difficult to validate our results in an independent cohort or to fully adjust for heterogeneity in patient backgrounds. Second, the lack of appropriate experimental models, such as eccrine sweat gland culture systems, prevented us from directly demonstrating the detailed IFN-γ–mediated immune responses specific to eccrine ducts. Third, the present study is primarily correlative and does not provide direct functional evidence. In addition, co-localization analyses using independent datasets such as a public spatial transcriptomic database were not performed, and this remains an important subject for future investigation. Nevertheless, the elevations of CCL22 and MIF observed in this study were consistently detected across patients with AIGA. These shared findings may therefore represent characteristic features of AIGA and provide meaningful insights into its immunopathology.

In conclusion, our study provides new insight into the immunopathogenesis of AIGA, highlighting the potential role of periductal macrophages and a CCL22–IFN-γ–CXCL10 axis in the recruitment and activation of autoreactive T cells. These findings not only contribute to the understanding of AIGA but also suggest that targeting CCL22 may represent a novel therapeutic approach.

## Data Availability

The raw data supporting the conclusions of this article will be made available by the authors, without undue reservation.
