# Supplementary figures and images for: CCL22-producing macrophages are associated with Th1-related sweat duct inflammation in acquired idiopathic generalized anhidrosis
Source: Front Immunol. 2026 May 8;17:1831853. doi: 10.3389/fimmu.2026.1831853 (PMC13194355; doi:10.3389/fimmu.2026.1831853)

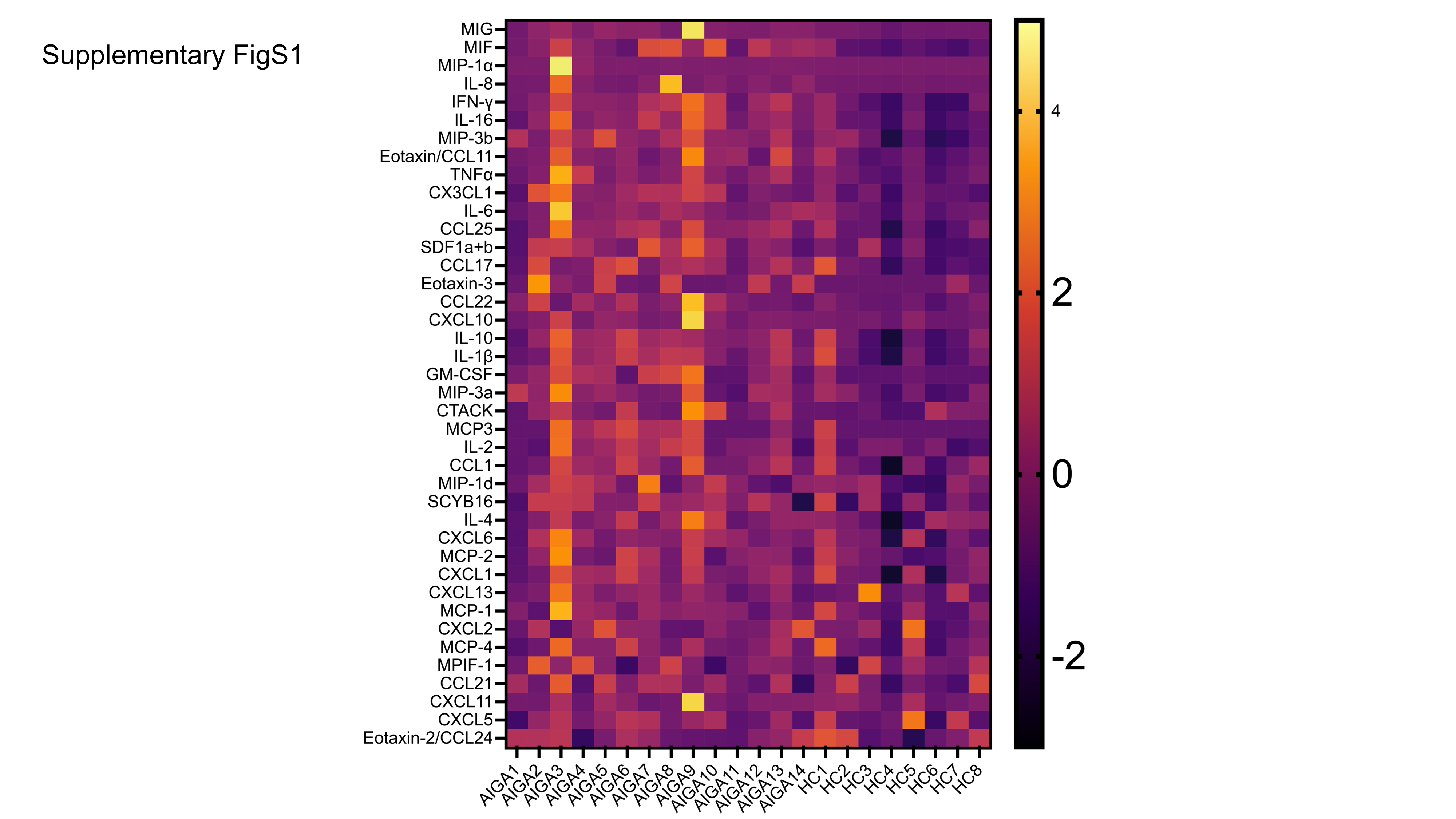

Supplement: Supplementary file 1 [file Image1.tif]

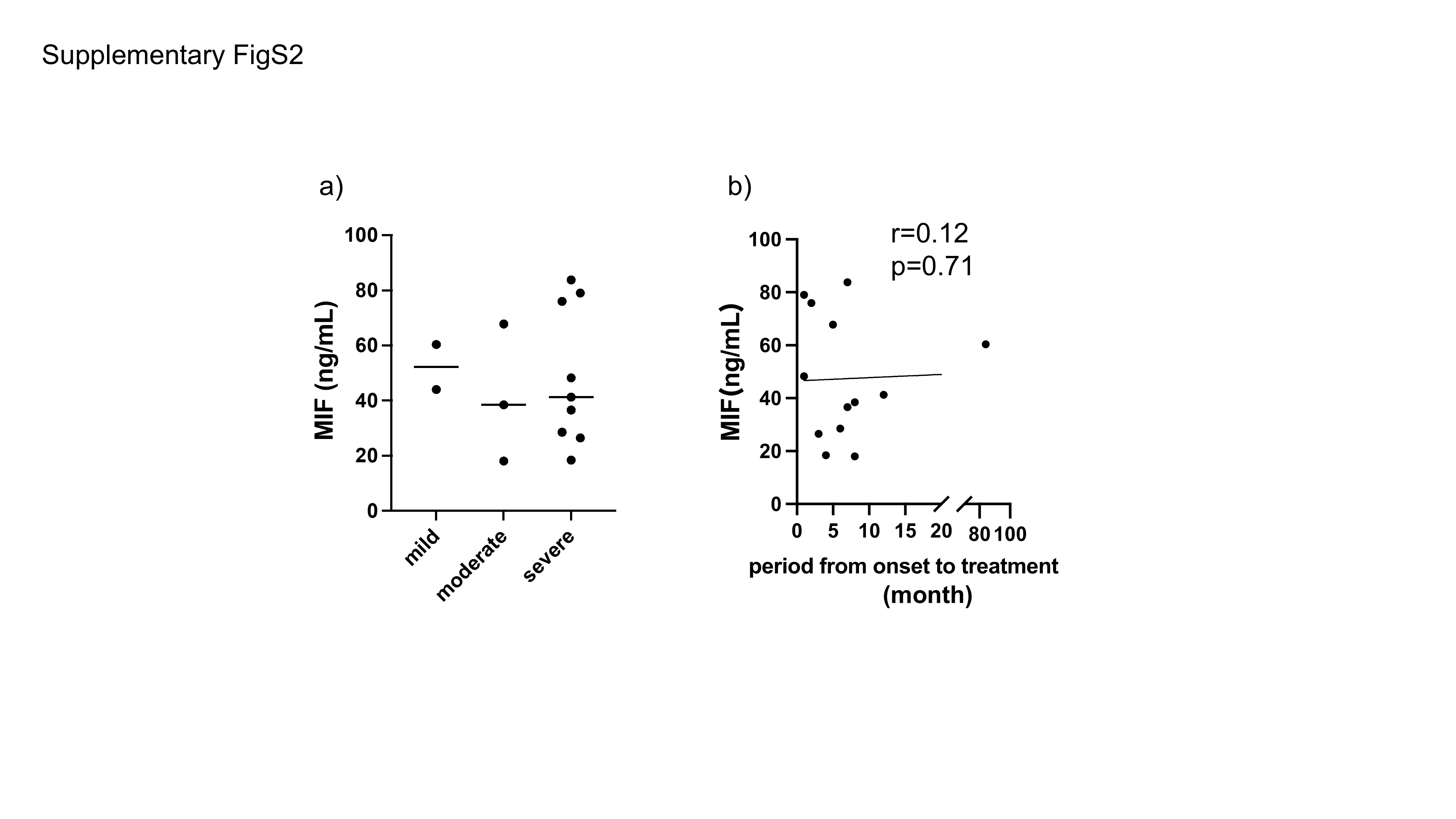

Supplement: Supplementary file 2 [file Image2.tif]

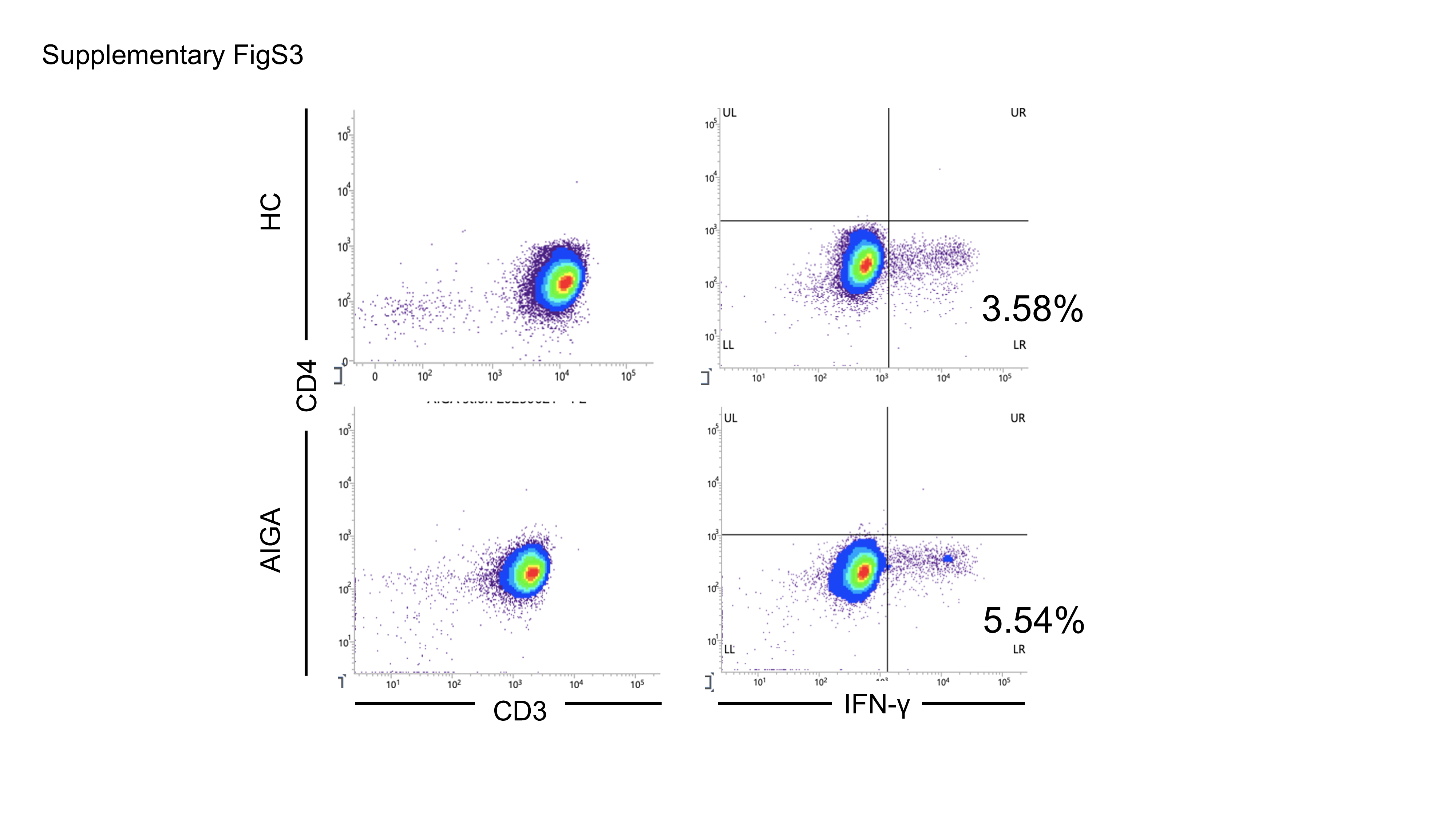

Supplement: Supplementary file 3 [file Image3.tif]

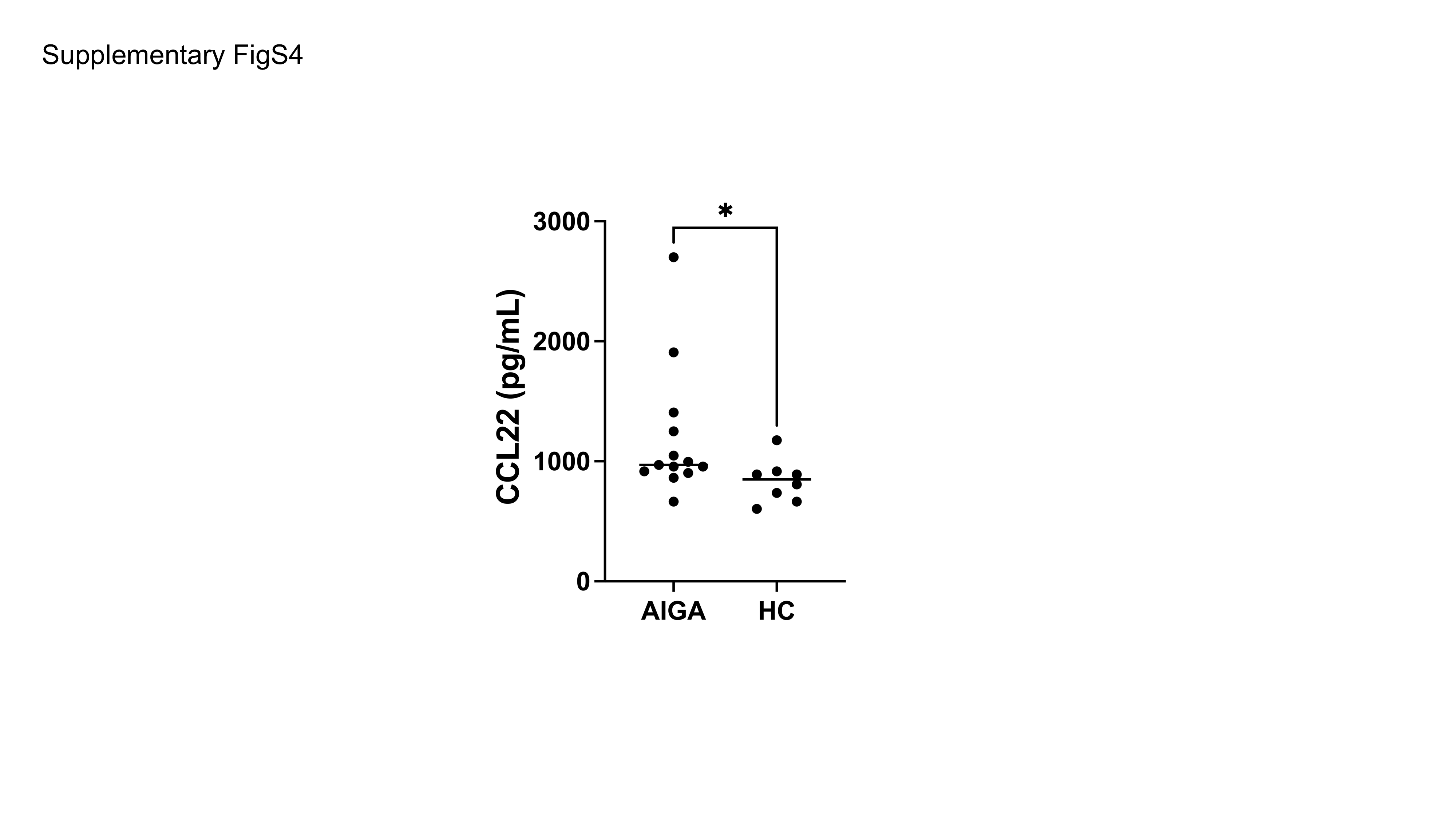

Supplement: Supplementary file 4 [file Image4.tif]

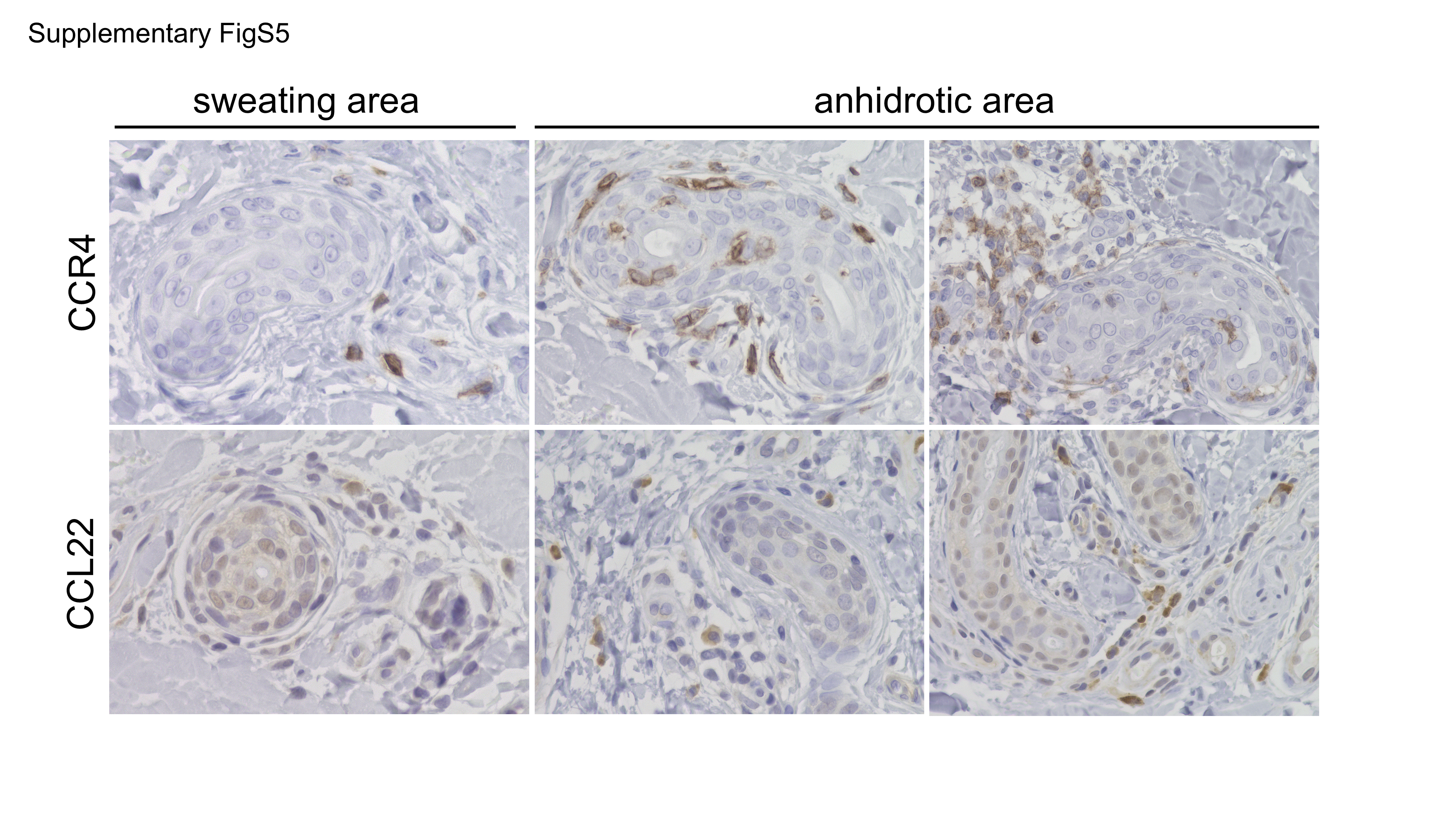

Supplement: Supplementary file 5 [file Image5.tif]
